# Supplementary material for: Targeting Galectin-3 to modulate inflammation in LAMA2-deficient congenital muscular dystrophy
Source: bioRxiv. 2025 Mar 14:2025.03.12.642905. Preprint. [Version 1] doi: 10.1101/2025.03.12.642905 (PMC11952532; doi:10.1101/2025.03.12.642905)
Supplement: 1 [file NIHPP2025.03.12.642905V1-supplement-1.pdf]

Supplemental Figure 1

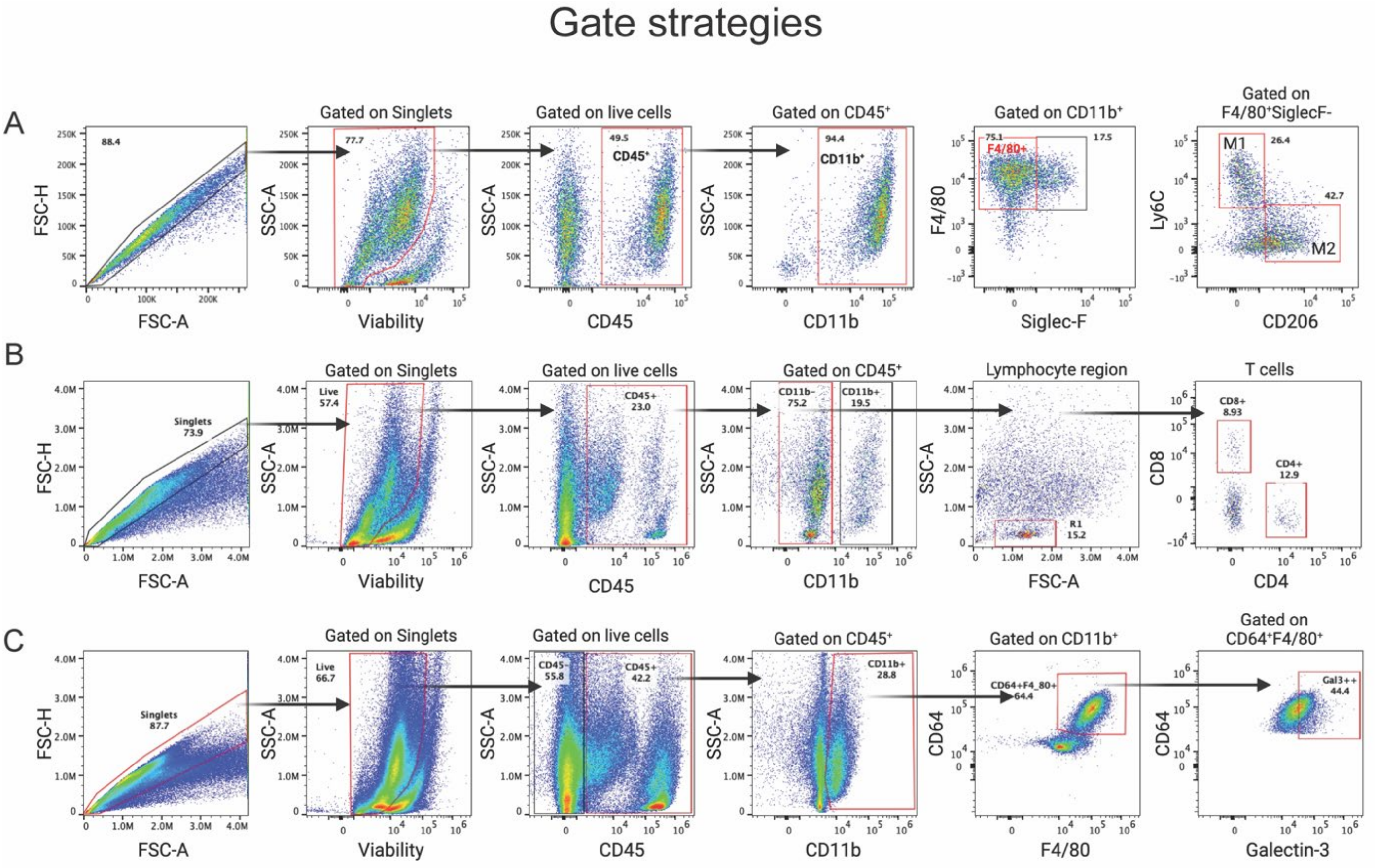

Supplemental Figure 1: Gating strategies used to identify targeted populations in the muscles. (A) Gating strategy for Macrophages. Single cell suspensions were gated based on their scatter to identify singlets followed by exclusion of dead cells. Leukocytes were identified based on CD45 staining. Myeloid cells were identified by CD45$^+$CD11b$^+$. Macrophages were identified by F4/80$^+$ and SiglecF$^-$ staining. M1 macrophages were identified as F4/80$^+$SiglecF$^-$Ly6C$^+$CD206$^-$ and M2 macrophages as F4/80$^+$SiglecF$^-$CD206$^+$Ly6c$^-$. (B) CD4 and CD8 T cells were identified by gating on CD11b$^-$ (negative) cells. (C) Galectin-3+ Macrophages were identified by the expression of CD11b$^+$CD64$^+$F4/80$^+$Galectin3$^+$ markers.

Supplemental Figure 2

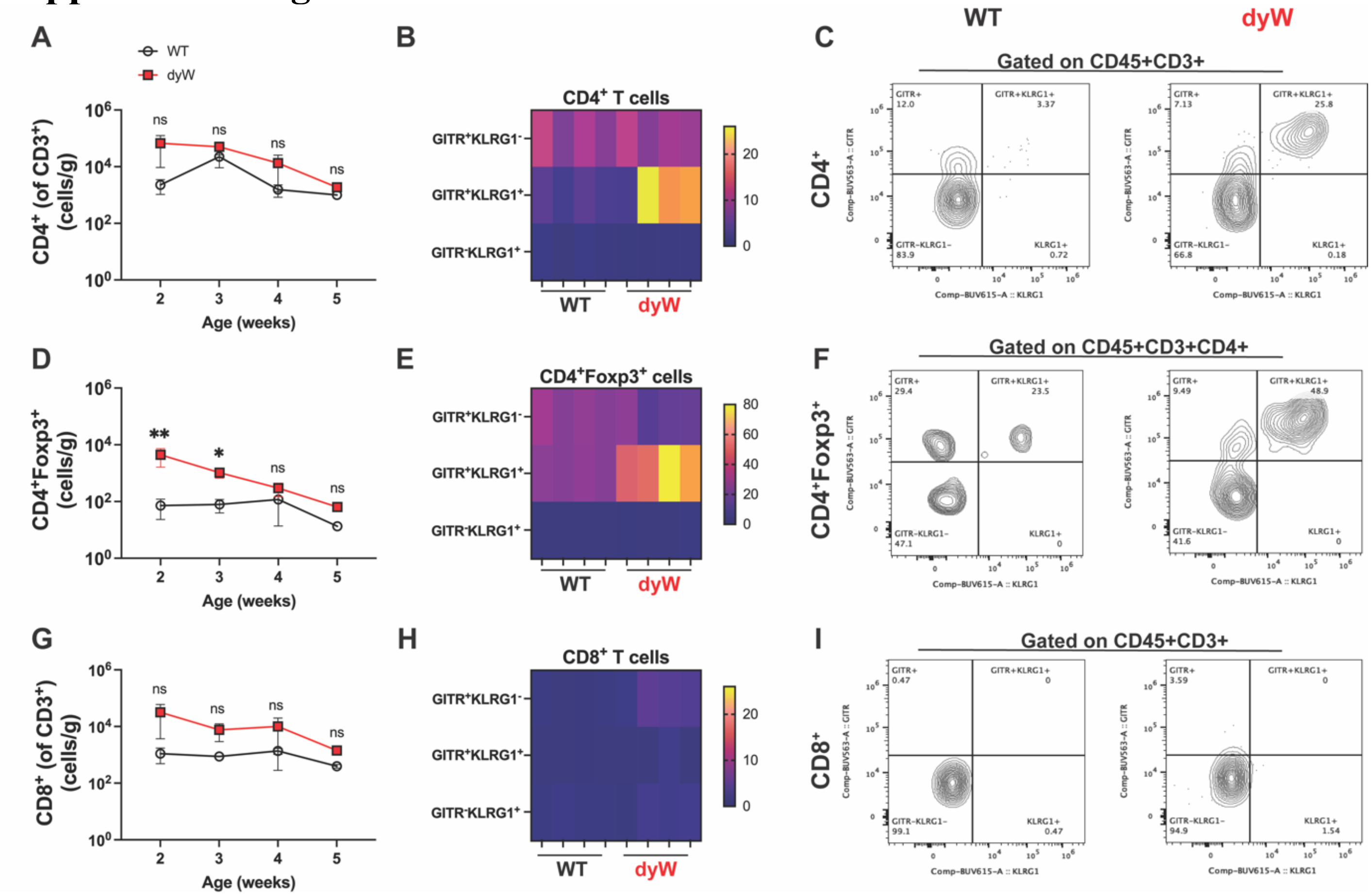

Supplemental Figure 2: Characterization of CD3+ T lymphocytes in dyW muscles. (A) Absolute CD4+CD3+ cell counts in WT and dyW mice. (B) Expression of activation markers GITR and/or KLRG1 at 2 weeks in CD4+ cells. (C) Representative dot plots showing increased frequency of CD4+ T cells expressing GITR+KLRG1 at 2 weeks. (D) Absolute Tregs CD4+Foxp3+ cell counts in WT and dyW mice. (E) Expression of activation markers GITR and/or KLRG1 at 2 weeks in Tregs. (F) Representative dot plots showing increased frequency of CD4+Foxp3+ cells expressing GITR+KLRG1 at 2 weeks. (G) Absolute Tregs CD8+CD3+ cell counts in WT and dyW mice. (H) Expression of activation markers GITR and/or KLRG1 at 2 weeks in CD8+ cells. (I) Representative dot plots showing the frequency of CD8+ cells expressing GITR and/or KLRG1 at 2 weeks. n= 5-7 mice/group. Data are presented as mean ± SEM. Statistical significance was determined by one-way ANOVA with Tukey's multiple comparisons test (ns= non-significant, *p<0.05, **p<0.01).

Supplemental Figure 3

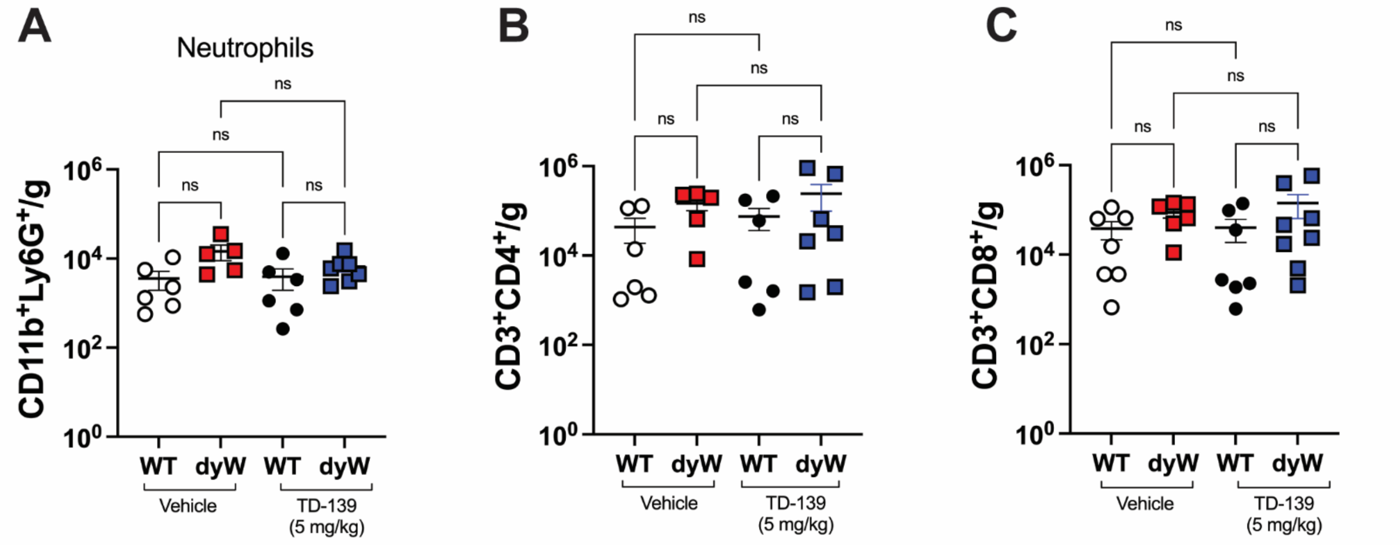

Supplemental Figure 3: TD-139 does not alter Neutrophils and T cells counts in the muscles. (A) Absolute live Neutrophils ($CD11b^{+}Ly6G^{+}$) counts in quadriceps from WT and dyW treated either with vehicle or TD-139. (B) Absolute live $CD4^{+}$ T cells and (C) $CD8^{+}$ T cells counts in quadriceps from WT and dyW treated either with vehicle or TD-139. n= 5-7 mice/group. The results are pooled from two independent experiments. Data are presented as mean ± SEM. Statistical significance was determined by one-way ANOVA with Tukey's multiple comparisons test (ns= non-significant).

Supplemental Figure 4

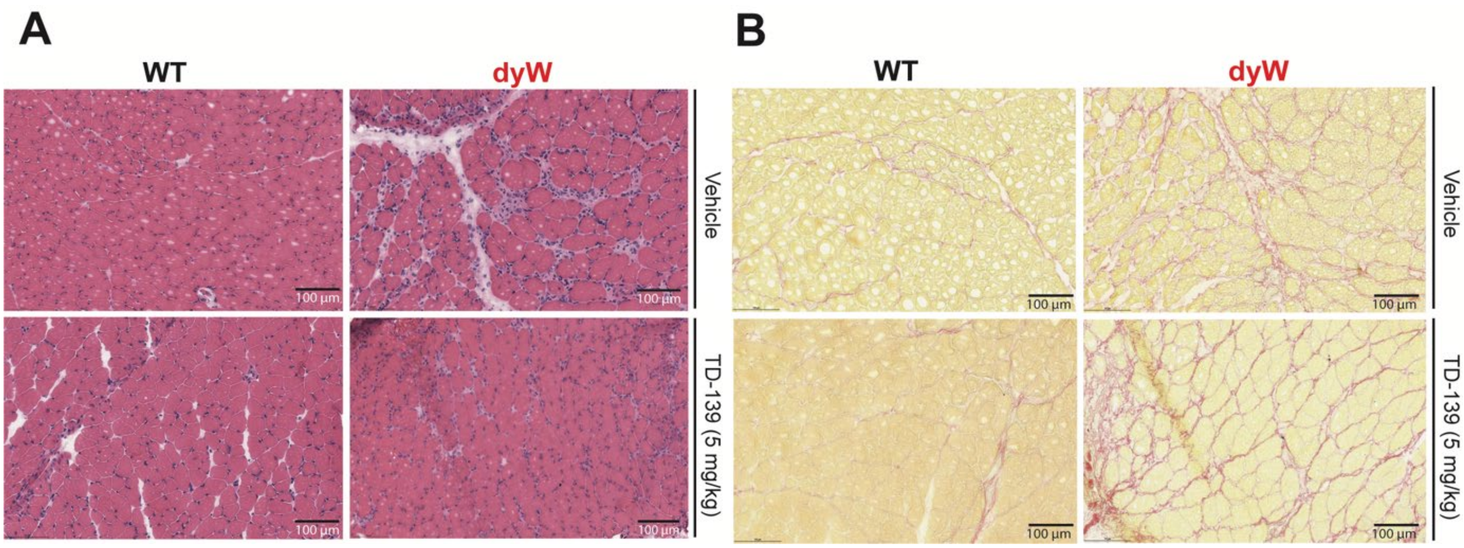

Supplemental Figure 4: Impact of TD-139 on muscle histopathology. **(A)** Representative H&E and **(B)** Picrosirius red staining images of quadriceps muscle frozen section from WT, dyW vehicle and TD-139 treated (scale bar 100 µm).
